# Aggressive angiomayxoma in men: Case report and systematic review

**DOI:** 10.1016/j.amsu.2022.103880

**Published:** 2022-06-15

**Authors:** Aziz Joseph Sabbagh, Khaled Arnaout, Ahmad Yamen Arnaout, Bayan Toutounji, Lina Ghabreau, Kusay Ayoub, Ibrahim Al-Hadid

**Affiliations:** a Department of Urosurgery, University of Aleppo, Aleppo, Syria; b Faculty of Medicine, University of Aleppo, Aleppo, Syria; c Department of Pulmonology, Faculty of Medicine, Aleppo University, Aleppo, Syria; d Department of Pathology, Faculty of Medicine, Aleppo University, Aleppo, Syria; e Instructor at General Surgery Department, Aleppo University Hospital, Aleppo University, Faculty of Medicine, Aleppo, Syria

**Keywords:** Aggressive angiomyxoma, Immunohistochemistry, Mesenchymal tumor, Systematic review, Hormonal treatment

## Abstract

**Introduction:**

Aggressive angiomyxoma is a rare benign mesenchymal tumor and occurs rarely in males. This study aimed to review all the cases of AAM in men in the English literature up to September 2020 and investigate the clinical, histochemical, and radiological characteristics of AAM and discuss the best treatment choices according to available data.

**Methods:**

A comprehensive search of the PubMed, Google Scholar, and Embase databases up to September 2020 was performed looking for reported cases of male patients with AAM. The search excluded articles in languages other than English, reported female cases, and superficial angiomyxoma cases.

**Results:**

Among the 97 patients, the mean age was 48.2 years with an incidence peak between 40 and 60 years. The sites commonly involved were the scrotum (42.3%). On ultrasound, the tumor was hypoechoic (85.7%) with a well-defined margin (100%), whereas on MRI, most cases were isointense on T1-weighted images (53.8%), and hyperintense on T2-weighted images (85.7%). Immunohistochemistry revealed that the tumor tended to be positive for vimentin (100%), CD34 (63.4%), ER (50%), and PR (53.3%) while S-100 showed 91% negativity. Wide and complete surgical excision was conducted in most cases (72%), and follow-up duration ranged from 1 month to 144 months with a recurrence rate of 11.8%.

**Conclusion:**

Although the occurrence of AAM is rare in men, consideration should be taken in the differential diagnosis of a mass in the genitourinary region. According to our review, the most decisive immunohistochemistry profile is the positivity of Vimentin and CD34 with the negativity of S-100. Although hormonal treatment is controversial, we suggest a novel algorithm for the management of aggressive angiomyxoma.

## Highlights

• Aggressive angiomyxoma is a rare benign mesenchymal tumor that occurs rarely in men compared to the woman.
• Less than 90 cases in men have been reported up to September 2020.
• Radiologic imaging play role in the initial approach and the follow-up.
• The most decisive immunohistochemistry profile is the positivity of Vimentin and CD34 with the negativity of S-100.
• This review presents a novel algorithm for the management of aggressive angiomyxoma.

## 1 Introduction

Aggressive angiomyxoma is a rare benign mesenchymal tumor that arises commonly from the soft tissue of the perineum and pelvis. It occurs commonly in females at the productive age with a male to female ratio of 1:6 [1,2]. The first case was reported by Steeper and Rosai in 1983, and two years later, the first case was reported in men. It commonly originates in males from the scrotum, inguinal region, and perineum, and has a high risk of local recurrence after surgical excision without metastasizing [1,3,4]. The term “aggressive” indicates the local infiltrative nature of the tumor and the high risk of local recurrence. Overall, the term “angiomyxoma” describes the nature of the histological characteristics that consist of myxoid with diffuse vascular disease [5,6].

Here we report on a rare clinical case of aggressive angiomyxoma in man and review systemically all cases in men up to September 2020.

## 2 Case presentation

A 72-year-old man presented to our out-patient clinic at Aleppo university hospital complaining of an increase in the size of the right testicle without pain, redness, or localized heat. Regarding his past medical history, he has had hypertension for 20 years, benign prostatic hypertrophy for 20 years, chronic hemorrhoids, and chronic laxative-dependent constipation. He has no past surgical history. Drugs history included aspirin 100 mg, Proton-pump inhibitors (PPIs) over ten years, Tamsulosin, herbal laxatives for 25 years, and neurotoxic medications Luxstan 1.5 mg. The patient is a smoker, smoking approximately 58 packets/year, and consumes alcohol approximately once a week for 50 years. The patient has no allergies or traumatic histories, and his family history was clear of malignancies. On clinical examination, there was palpation of a hard, moving, painless mass above the right testicle.

Laboratory tests showed a mildly raised level of serum glucose, but other biochemical and hormonal tests were normal. Beta-human chorionic gonadotropins (B-HCG), alpha-fetoprotein, and prostate-specific antigen (PSA) markers were also within normal limits. A scrotal ultrasound showed a well-defined, oval, hypoechoic, homogeneous tissue above the right testis measuring 6.1 × 4.5 cm with internal vascularity. It squeezes the medial part of the right epididymis head [Supplementary Figure 1]. Our surgical team performed radical orchiectomy.

Macroscopically, a testis measuring 5 × 6.5 × 9 with a spermatic cord. Cut section showed para-testicular well-defined mass measuring 6.5 cm with a greater diameter. Microscopically, a poorly circumscribed infiltrative tumor made up of bland, spindled, and stellate cells with delicate cytoplasmic processes that surrounded blood vessels, on the myxoid stroma. There were no atypia or mitotic figures. Immunohistochemically, tumor cells were positive for estrogen receptors (ER), progesterone receptors (PR), and focally for smooth muscle actin (SMA), while negative for calretinin, CD34, desmin, and S-100 protein. Additionally, Ki67 proliferation marker was low (<5%) [Supplementary Figure 2]. The final diagnosis was then made as aggressive angiomyxoma with clear resection margins.

Multislice computed tomography (CT) scan for chest, abdomen, and pelvic was performed ten days after surgery and showed no metastases, lymphadenopathy, or free fluids. Two years postoperatively, the patient was fine without signs of recurrence, as determined by magnetic resonance imaging (MRI) and CT scan.

## 3 Material and methods

### 3.1 Search strategy

**Fig. 1:**
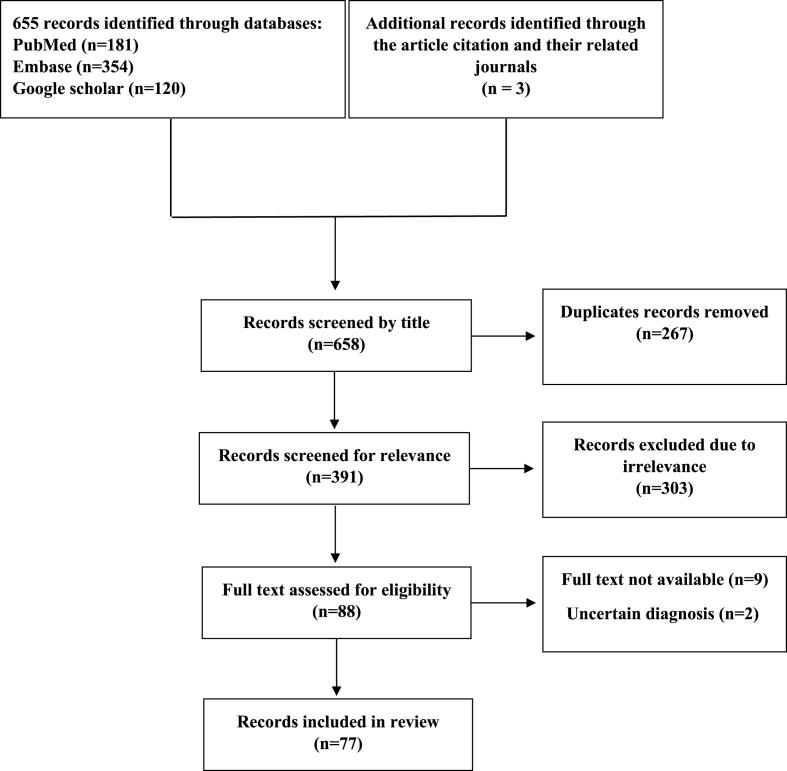
PRISMA flow diagram for the study.

A literature search of computerized medical literature was performed using the PubMed, EMBASE, and Google Scholar. The keywords searched were (“angiomyxoma” OR “aggressive angiomyxoma”) AND (“Male"[Mesh] OR “Genital Neoplasms, Male"[Mesh] OR “Genitalia, Male"[Mesh] OR “Male Urogenital Diseases"[Mesh]). The search terms were modified to fit each database. The search was conducted for published papers up to September 2020, and restricted to the English language. To ensure that the search was complete, reference lists and similar articles were manually searched to identify additional relevant studies. We conducted our systematic review following to recommendations of Preferred Reporting Items for Systematic Reviews and Meta-Analyses 2020 (PRISMA 2020) [Fig. 1] and it is registered in OSF Registries with registration DOI**:** 10.17605/OSF.IO/U64WB**.** In addition, the case report has been prepared and reported in line with the SCARE guidelines [7].

### 3.2 Inclusion and exclusion criteria

Studies were included of cases involved angiomyxoma in male adults and children in the reproductive tract, pelvis, perineum, or extra-genitourinary region. Additionally, if the type of case report, case series, or any literature review contained a case report the study was included. Articles that had female cases, were in languages other than English, or involved superficial angiomyoxoma were excluded.

### 3.3 Screening, data collection and data analysis

Screening and data extraction was done by two review authors (AYA and BT) using the Rayyan tool for screening and Microsoft Excel 2010 for data extraction, and was checked by a third author (KhA) to resolve any disagreement. Data was analyzed using IBM SPSS Statistics 24**.**

## 4 Results

The first case in men was reported by Bégin et al., in 1985. Up to September 2020, 88 cases in men were reported with two peaks of publishing in 2008 and 2017. Among the 97 patients, the age of presentation varied from 9 months to 81 years (mean [SD] 48.24 [19.76] years; median, 50 years) with incidence peak between 40 and 60 years. The genitourinary region was commonly involved (85.6%), and the scrotum was the most common region compare to the extra-genitourinary region (14.4%) [Table 1]. The maximum diameter of the tumor ranged from 0.5 cm to 34 cm (mean [SD] 9.79 [7.17] cm; median, 7 cm). The clinical presentation and the physical examination varied according to the site and size of tumor [Supplementary Table 1].

**Table 1:** Summary of basic characteristics of the patients.

	Number of patients (%)
**Age of presentation:**	
<20	14/97 (14.4)
21–40	15/97 (15.5)
41–60	36/97 (37.1)
61–80	30/97 (30.9)
>80	2/97 (2.1)
**Site of Tumor:**	
Genitourinary region	83/97 (85.6)
Scrotum	41/97 (42.3)
Pelvis	11/97 (11.3)
Perineum	10/97 (10.3)
Groin	9/97 (9.3)
Spermatic cord	5/97 (5.2)
Prostate	4/97 (4.1)
Scrotum, penis	2/97 (2.1)
Penis	1/97 (1)
Extra-genitourinary region	14/97 (14.4)
Larynx	2/97 (2.1)
lower extremity	2/97 (2.1)
Orbit	2/97 (2.1)
Abdominal wall	1/97 (1)
Glabella	1/97 (1)
Greater Omentum	1/97 (1)
Jejunum	1/97 (1)
Maxilla	1/97 (1)
Sphenoidal Sinus	1/97 (1)
Supraclavicular Fossa	1/97 (1)
renal allograft	1/97 (1)
**Surgical Approach:**	
Surgical excision	37/93 (39.8)
Wide surgical excision	19/93 (20.4)
Complete surgical excision	12/93 (12.9)
Local surgical excision	10/93 (10.8)
Excisional Biopsy	6/93 (6.5)
Intralesional surgical excision	4/93 (4.3)
Endoscopic endonasal excision	1/93 (1.1)
Incisional biopsy	1/93 (1.1)
Subtotal surgical excision	2/93 (2.2)
Transcranial orbitotomy surgical excision	1/93 (1.1)
**Recurrence:**	
Number of reported recurrence patient	8/68 (11.8)
The period until the first recurrence (months)	(28.86, 2–84)
Follow-up (months)	(24.23, 1–144)

The tumor was hypoechoic on US (85.7%; n = 12/14) with well-defined margins (100%; n = 13/13) and no difference in internal echogenicity. On MRI, the most cases were isointense on T1-weighted images (53.8%; n = 7/13) and hyperintense on T2-weighted images (85.7%; n = 12/14). Regarding immunohistochemistry, the tumor tended to be positive for vimentin (100%; n = 35/35), CD34 (63.4%; n = 26/41), ER (50%; n = 16/32), and PR (53.3%; n = 16/30), while S-100 showed 91% negativity (n = 41/45). Alcian blue, Factor VII, and androgen receptor were 100% positive but present in the minority of cases. Fifteen cases out of sixteen were positive for Ki67 receptors with less than 10% and only one case out of sixteen had a 15% positivity percentage of the cells [Table 2].

**Table 2:** Summary of the radiologic and immunohistochemistry characteristics.

	Number of Cases (%)	
**Overall appearance – US**		
Hypoechoic	12/14 (85.7)	
Hyperechoeic	1/14 (7.1)	
Mixed echogenic	1/14 (7.1)	
**Margin - US**		
Well-defined	13/13 (100)	
**Internal echogenicity - US**		
Heterogeneous	10/19 (52.6)	
Homogeneous	9/19 (47.4)	
**TI-weighted - MRI**		
Isointense	7/13 (53.8)	
Hypointense	5/13 (38.5)	
Isohypointense	1/13 (7.7)	
**T2-weighted - MRI**		
Hyperintense	12/14 (85.7)	
Hypointense	1/14 (7.1)	
Isohyperintense	1/14 (7.1)	
**Immunohistochemistry profile:**	No. of positivity cases (%)	No. of negativity cases (%)
Vimentin	35/35 (100)	–
CD34	26/41 (63.4)	15/41 (36.6)
MSA	6/13 (46.2)	7/13 (53.8)
SMA	18/44 (40.9)	26/44 (59.1)
Desmin	25/54 (46.3)	29/54 (53.7)
S100	4/45 (8.9)	41/45 (91.1)
Alcian blue	–	1/1 (100)
Factor VII	–	1/1 (100)
Androgen receptor	5/5 (100)	–
Estrogen receptor (ER)	16/32 (50)	16/32 (50)
Progesterone receptor (PR)	16/30 (53.3)	14/30 (46.7)
Ki67 (<10%)	15/16 (93.8)	
Ki67 (10–20%)	1/16 (6.3)	

The surgical approach varied, wide and complete surgical excision was conducted in the most cases (72%; n = 67/93), while frequency of local surgical excision and excisional biopsy was (17.4%; n = 16/93). Four patients were treated with hormonal treatment, and in two of them, the tumor was positive for estrogen and progesterone receptors. Radiotherapy was conducted in 2 patients and chemotherapy in 1 patient. Recurrence rate was 11.8% (n = 8/68) and the mean time period for recurrence was 28.8 months. The minimum time period until recurrence was 2 months, while the maximum time period was 84 months. Follow-up duration ranged from 1 months to 144 months [Table 1].

## 5 Discussion

AAM is a benign mesenchymal tumor that arises mostly from the soft tissues of the pelvic region in premenopausal women, which was first reported in 1983 by Steeper and Rosai, and 2 years later, the first case in a man was described by Bégin. AAM shows female predominance, as less than 90 male cases have been reported to date [1,2,4,8]. In men, the tumor originates commonly from the scrotum (42%), pelvis (11%), perineum (10%), groin (9%), spermatic cord (5%), and prostate (4%). Only 3 cases reported a tumor in the penis and scrotum extended to the penis. Tumors also could appear in rare extra-genitourinary regions, including extremities, the neck, and the head. For example, one case described the tumor in a renal allograft. In men, AAM often occurs in the middle-ages, with a peak incidence between 40 and 80 years (68%), and a mean age of 48.2 years [Table 1]. Only 11 cases reported child patients below 18 years, and the youngest reported case was a 9 month old child [9]. The clinical presentation of AAM varies according to the site and size of the lesion, but commonly presents as painless swelling over a specified time in the scrotum. Misdiagnosis can occur, being documented as testicular neoplasm, inguinal hernia, hydrocele, or spermatocele. AAM in the prostate mimics the signs and symptoms of prostate enlargement and causes lower urinary symptoms, while in other regions presents with the symptoms of mass that compress the neighboring organs. Physical examination in most cases reveals a firm, non-tender, and non-transilluminated mass [Supplementary Table 1].

Imaging studies play an important role to make the diagnosis preoperatively when the malignancy is suspected. On US, the tumor revealed a hypoechoic, heterogeneous mass with a well-defined margin. However, one case showed a hyperechoic mass. MRI provides more advantages in identifying the characteristics of such mesenchymal tumors, including isointense or hypointense characteristics on T1-weighted images and hyperintense characteristics on T2-weighted images. One case showed hypointensity on the T2-weighted image and another iso-hyperintensity. The swirled sign observed on both US and MRI has also been mentioned in a recent study [10]. Typical CT features are a mass hypodense or isodense to muscle with well-defined margins that show variable enhancement after administration of intravenous contrast [11]. Considering CT appearance is unspecific, the suspected diagnosis must be based again on the characteristic morphology and localization of the mass, and should be complemented with MRI. However, none of these radiologic investigations can discriminate AAM from different malignancies, including sarcomas [12]. Without histological and immunohistochemical studies, there could be a preoperative misdiagnosis rate of 70%–100% [13].

Histologically, aggressive angiomyxomas consist of mesenchymal spindle-shaped or stellate cells embedded in a myxoid matrix with abundant fibroblasts, myofibroblasts, and variably sized vessels. Angiomyxomas show low mitotic activity with an absence of nuclear atypia [8], which corresponds with our histology. Regarding immunohistochemistry, there is no definitive stain to date that can make the final diagnosis alone, but an immunohistochemistry profile may strongly contribute to the diagnosis. The most decisive immunohistochemistry profile, according to our review, is the positivity of Vimentin and CD34 with the negativity of S-100. MSA, SMA, and Desmin are of medium sensitivity and specificity because they reported positivity in about 50% of the cases. Stunningly, ER and PR receptors reacted positively in 50% of cases (n = 32), even when the tumor was in men. This proposes a hormonal effect on the tumor growth and may arise from specialized cells of the stroma of the perineum. Thus, hormonal therapy might be useful as an adjuvant treatment. This also may clarify why these tumors occur most frequently during reproductive age. However, a negative reaction for ER and PR receptors have been reported in two 13 year old teenagers, and one case reported positivity for ER and PR receptors in an 11 year old child [14,15]. Five cases expressed a positive reaction to androgen receptors, therefore, we need to analyze more studies to determine the relation between the tumor and hormonal factors. Fifteen cases out of sixteen revealed a positive expression of Ki67 in less than 10% of the tumor cells, and only one case revealed positivity in 15% of tumor cells confirming that this tumor is benign [Table 2].

Wide surgical excision is the treatment of choice, including broad free margins around the tumor in the area of resection. This should be the first step in preventing recurrences of these lesions, although it is dependent on tumor size, location, and the presence or absence of disease within the surgical margin. However, a literature review revealed no differences in recurrence between the patients with negative margins and those with positive margins [16]. These results may be explained by the locally infiltrative nature of AAM. As this tumor may express ER and PR receptors, hormonal neoadjuvant or adjuvant therapy have been used in woman with AAM, and has accomplished a decrease in tumor size in several reported cases [17,18]. These results establish a future type of therapy for such benign tumors. Four cases in men reported using hormonal therapy with gonadotropin-releasing hormone (GnRH) agonist either as adjuvant therapy to prevent recurrence or to manage recurrence prior to recurrence surgery [[19], [20], [21], [22]]. Additionally, many studies have showed promising results of using a single monthly 3.75 mg injection of leuprolide acetate, an agonist of (GnRH), for treatment of these lesions in women. In these studies, lesions measured clinically and with imaging, either disappear or decrease in size within three months to a year. Results depend on the size of the lesion and whether it is a primary or secondary tumor [17]. Another reported neoadjuvant therapy, using aromatase inhibitors, performed prior to surgery has also had success in reducing the size of the tumor [23]. However, whether an androgen agonist could be used as hormonal therapy in men with AR positivity needs to be further investigated [18].

The combination of the low metastasizing potential in AAM cases and the low mitotic activity of the tumor cells, chemotherapy and radiotherapy will have an inefficient role in adjuvant treatment [17]. However, Hidayat et al. [9] reported an AAM case in orbit that was treated with methotrexate following the surgery, because the whole tumor could not be resected due to infiltration in the cranial nerves and sinus. Additionally, two cases in men were reported using radiotherapy as possible adjuvant treatment post-surgery although of the benignity of the tumor and the low mitotic activity. In one case, the cause was the misdiagnosis of the tumor as myxolipoma and subsequent mistreatment. This proceeded until three recurrences occurred and finally a correct diagnosis of AAM in the leg was made. At this point, the tumor infiltrated the deep fascia of leg and the surgery would have left a large functional defect, therefore the patient was referred to the radiotherapy department for possible adjuvant [23,24].

Studies have referred to a high local recurrence rate between 36% and 72%, but no metastasis has been reported to date in men [24]. However, according to our review, the recurrence rate is 11.8% (n = 8/68), and the mean time period until the first recurrence is 28.8 months, with a range of 2–84 months. Follow-up duration ranged from 1 to 144 months and the MRI is the preferred method for detecting recurrences [3]. Metastases are exceedingly rare, and overall, the prognosis is good [25].

Although we conducted a systematic review of all cases reported in the medical literature on this topic, there are limitations to our study. For example, the available evidence remains weak because they all are case reports or series. In addition, we did not evaluate accepted studies and collected results without evaluating the evidence, but we excluded studies that did not accurately explain the method of diagnosis. However, our study remains the best available evidence about this tumor in men.

## 6 Conclusions

**Fig. 2:**
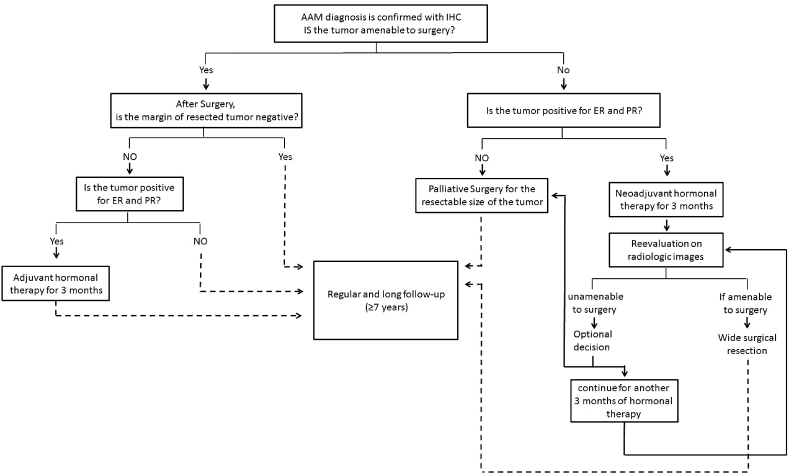
The suggested algorithm for management of aggressive angiomayoxoma (AAM) – IHC (Immunohistochemistry).

Although the occurrence of AAM is rare in men, consideration should be taken in the differential diagnosis of a mass in the genitourinary region. Radiologic images play a role in the initial approach, but an accurate diagnosis is made by the histochemistry and immunochemistry studies. According to our review, the most decisive immunohistochemistry profile is the positivity of Vimentin and CD34 with the negativity of S-100. In terms of treatment, wide surgical excision continues to be the standard. Although hormonal treatment is controversial, based on the available data and previous reports, we suggest a novel algorithm for the management of aggressive angiomyxoma (Fig. 2).

## Ethical approval

There is no need for ethical approval.

## Sources of funding

None.

## Author contributions

**Aziz Joseph Sabbagh** performed the surgery, followed the patient, and made a substantial contribution to the conception and design, drafting, and revision of the article.

Khaled Arnaout conducted a literature search, data analysis, and made a substantial contribution to the interpretation of data, drafting, and revision of the article.

**Aziz Joseph Sabbagh** and Khaled Arnaout contributed equally to this paper as a co-first author.

**Ahmad Yamen Arnaout** and **Bayan Toutounji** made a substantial contribution to conception and design, screening the articles, extracting the data, drafting, and revision the article.

**Lina Ghabreau** performed the immunohistochemical staining, interpreted the data, and critically revised the article.

**Kusay Ayoub** supervised the study, interpreted the data, and critically revised the article.

**Ibrahim Al-Hadid** supervised the study and the surgery, interpreted the data, and critically revised the article.

All authors read and approved the final version of the manuscript.

## Consent

Written informed consent was obtained from the patient for publication of this case report and any accompanying images.

## Registration of research studies

1. Name of the registry: OSF Registries.

2. Unique Identifying number or registration ID: 10.17605/OSF.IO/U64WB.

3. Hyperlink to your specific registration (must be publicly accessible and will be checked): https://archive.org/details/osf-registrations-u64wb-v1.

## Guarantor

Ahmad Yamen Arnaout. Yamen.arnout@gmail.com.

## Provenance and peer review

Not commissioned, externally peer reviewed.

## Declaration of competing interest

None.

## Appendix A Supplementary data

Multimedia component 2 Multimedia component 2

Multinedia component 3 Multinedia component 3

Multinedia component 4 Multinedia component 4
