# Supplementary material for: Aggressive angiomayxoma in men: Case report and systematic review
Source: Ann Med Surg (Lond). 2022 Jun 15;79:103880. doi: 10.1016/j.amsu.2022.103880 (PMC9289231; doi:10.1016/j.amsu.2022.103880)
Supplement: Multimedia component 2 [file mmc2.docx]

**Aggressive Angiomayxoma in Men: Case Report and Systematic Review**

**
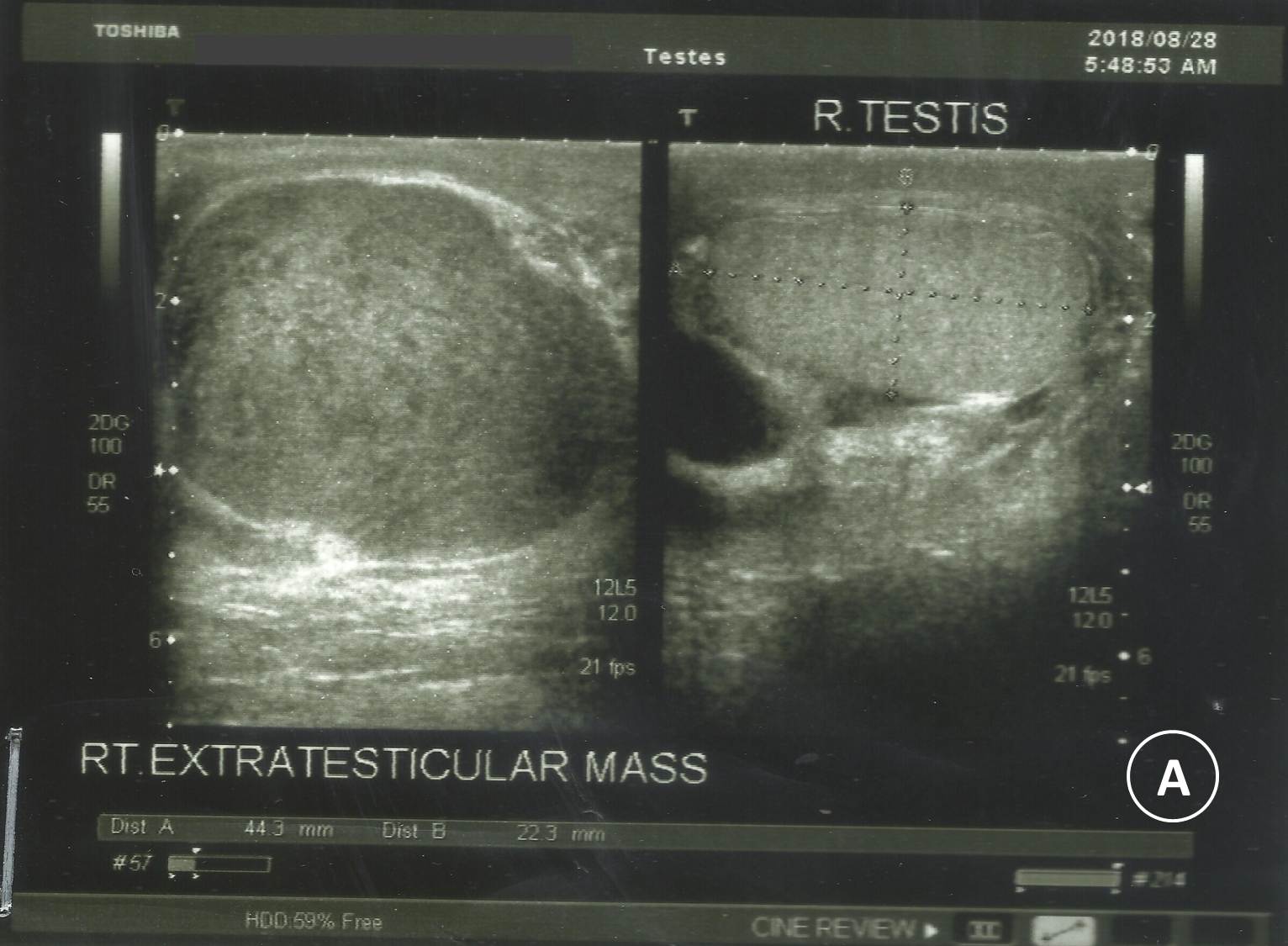
**

**
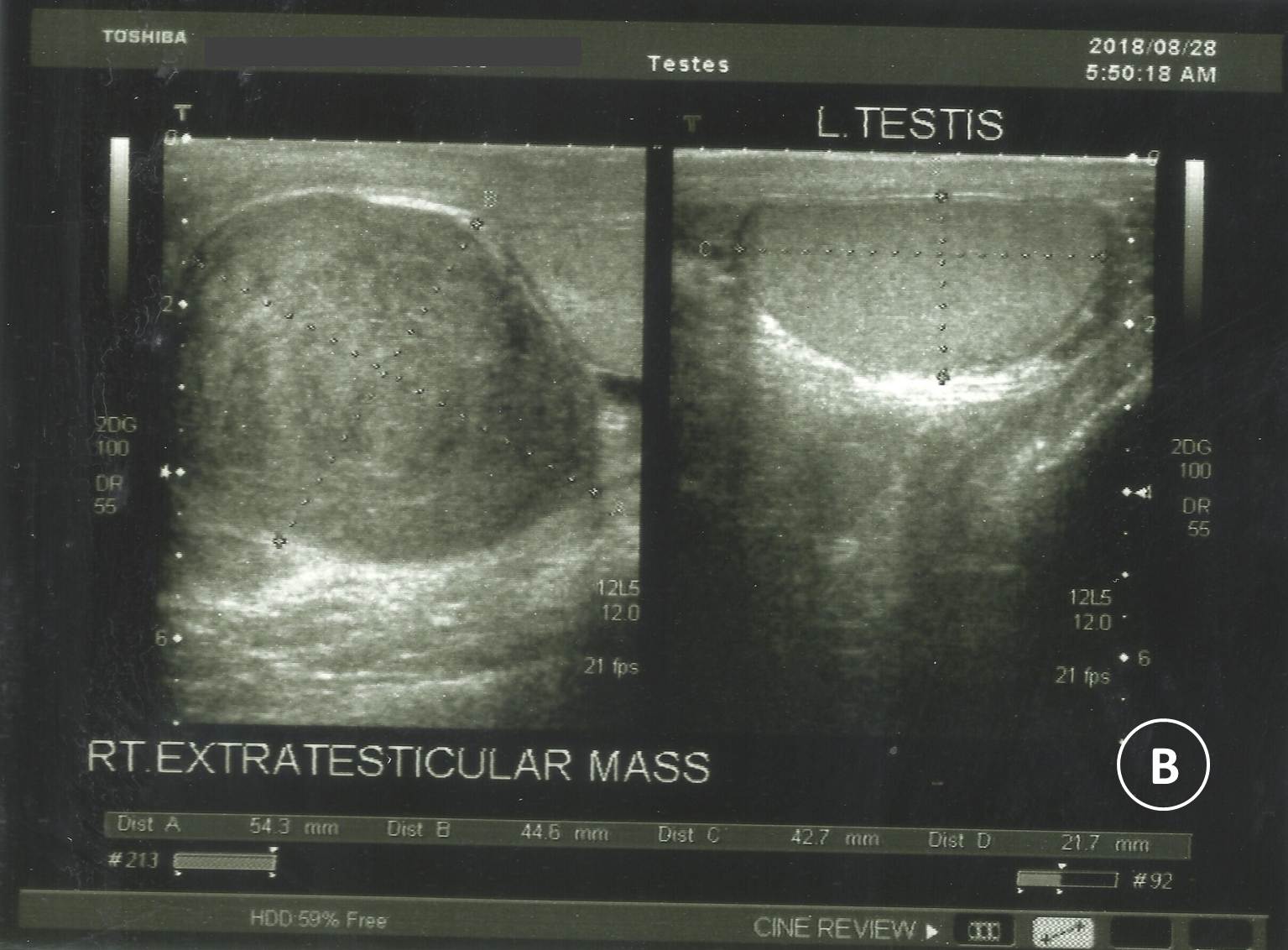
**

**
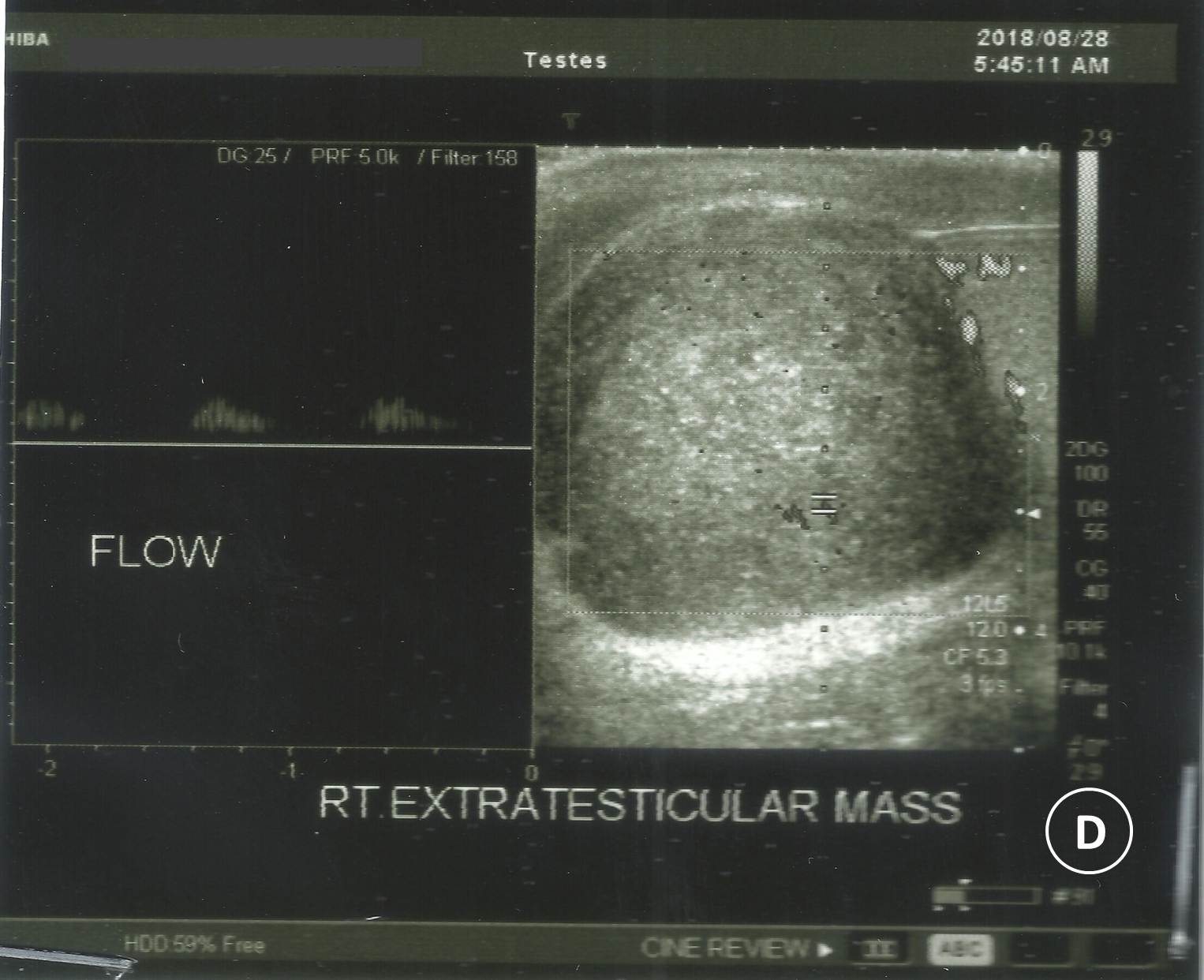

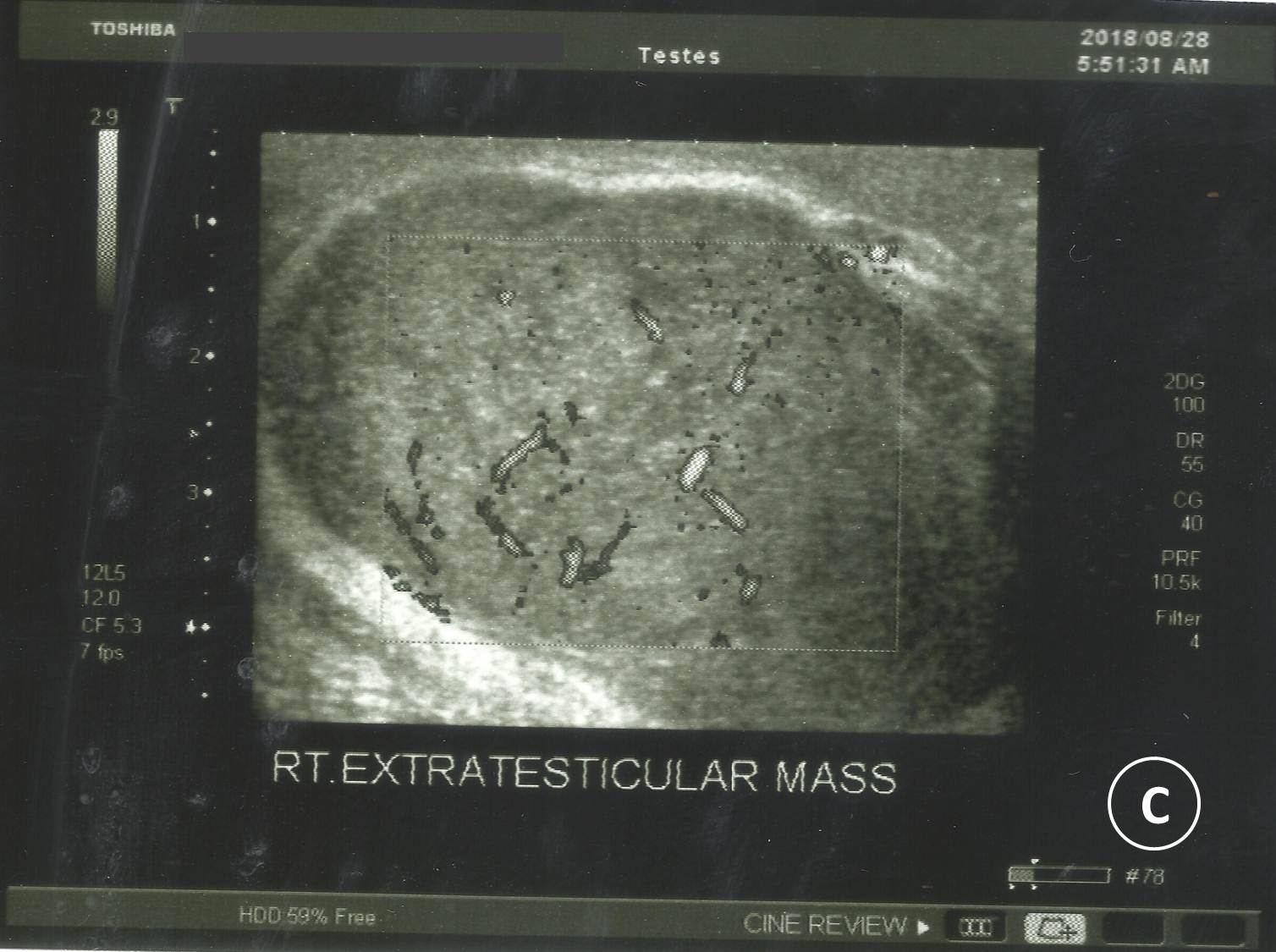
**

**
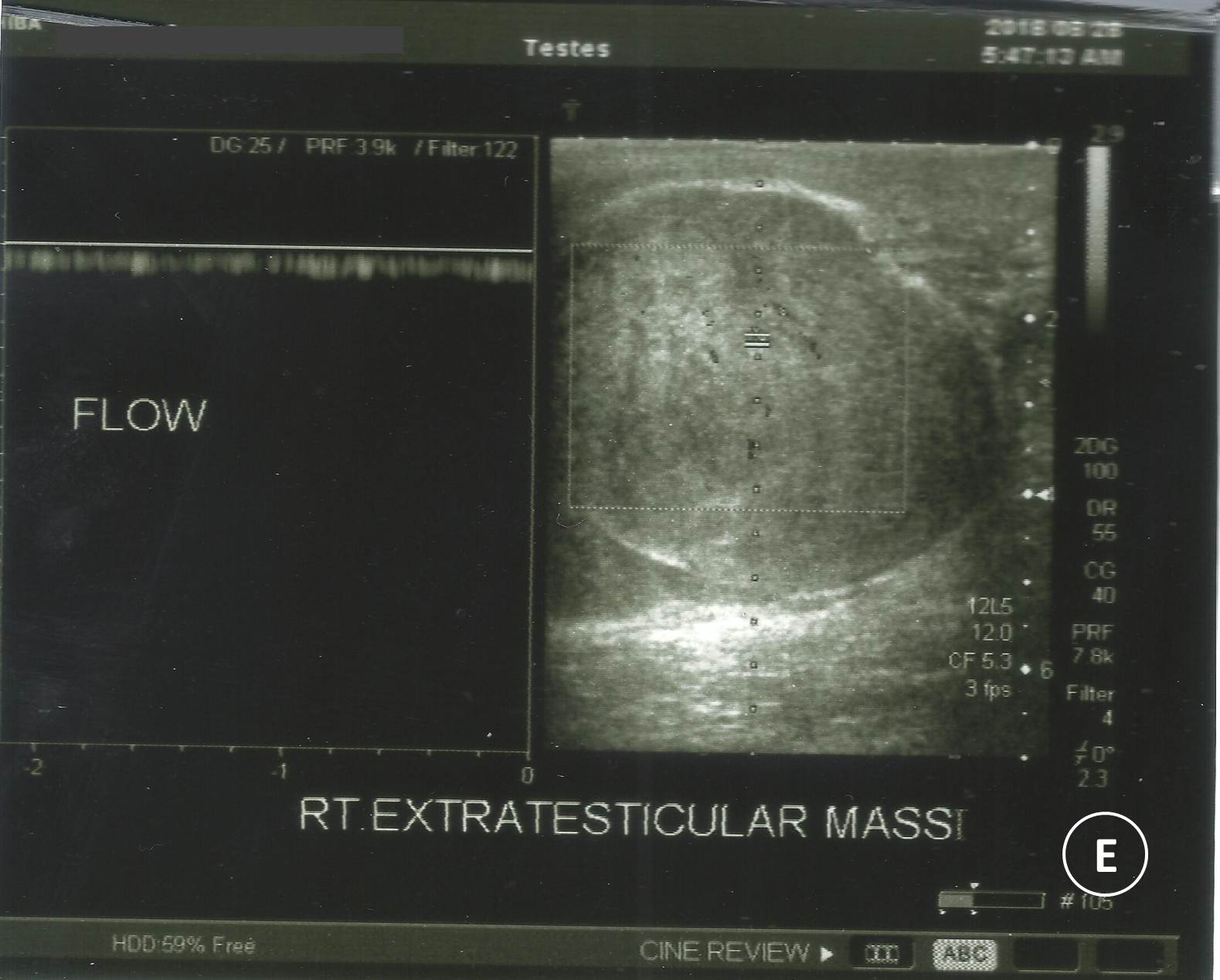
**

**Supplementary Figure 1:** Ultrasound showing (A) right testis (4.4*2.2 cm), (B) left testis (4.3*2.2 cm), and (C, D, E) right extra-testicular, well-defined, oval, and hypoechoic mass (6.1*4.5 cm) and homogeneous with internal vascularity.
