# Supplementary material for: Aggressive angiomayxoma in men: Case report and systematic review
Source: Ann Med Surg (Lond). 2022 Jun 15;79:103880. doi: 10.1016/j.amsu.2022.103880 (PMC9289231; doi:10.1016/j.amsu.2022.103880)
Supplement: Multinedia component 3 [file mmc3.docx]

**Aggressive Angiomayxoma in Men: Case Report and Systematic Review**

**
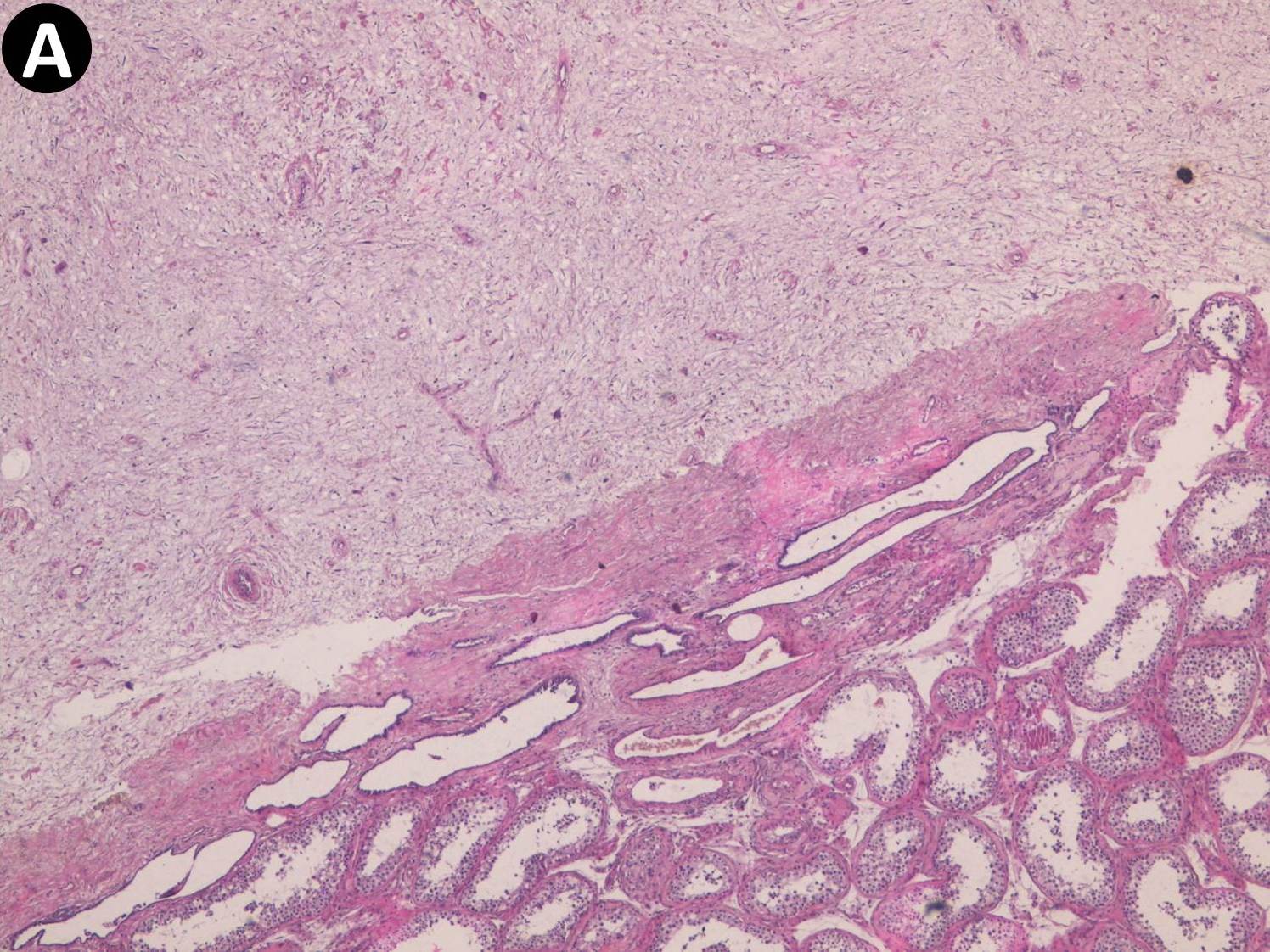
**

**
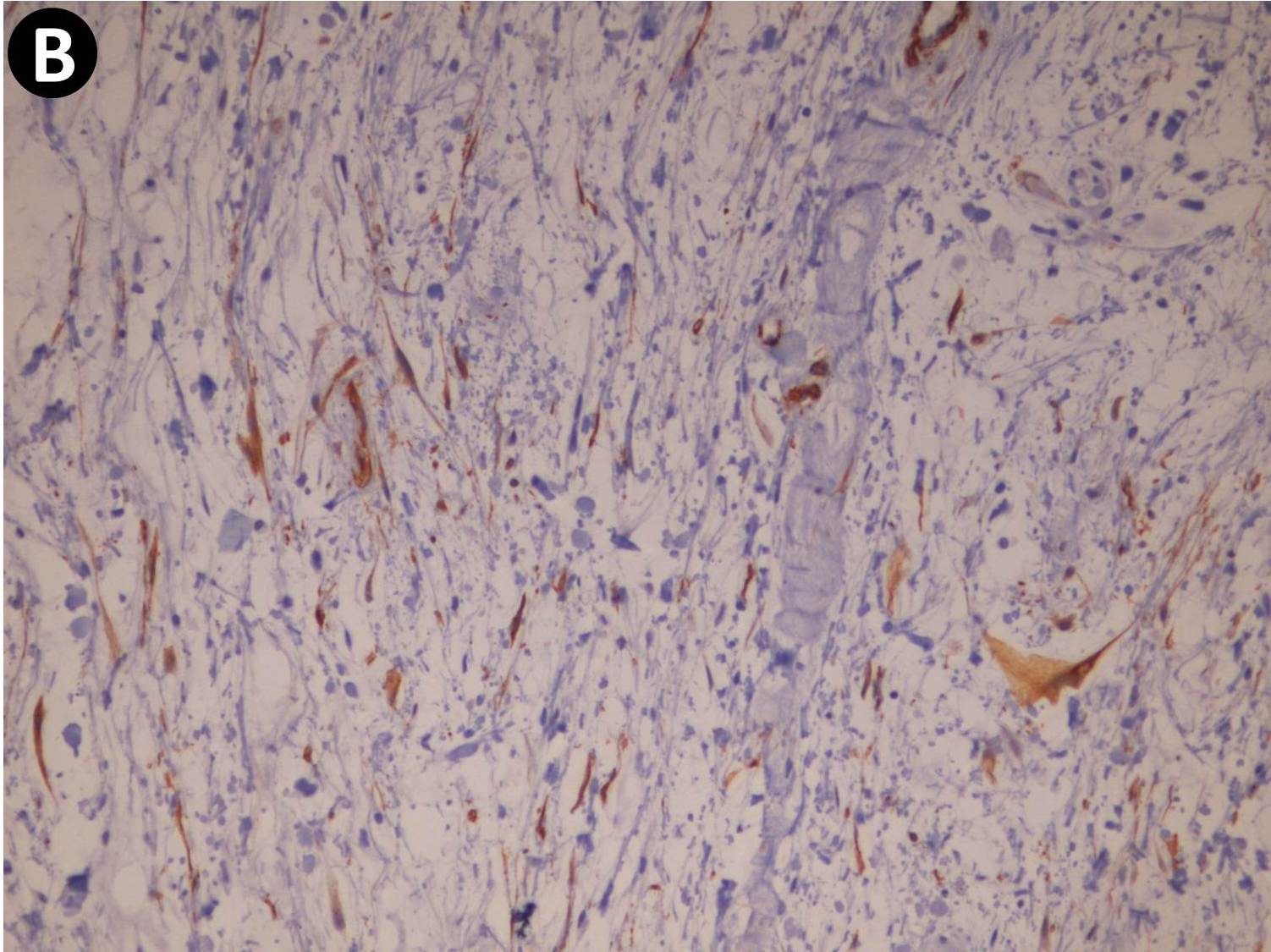
**

**
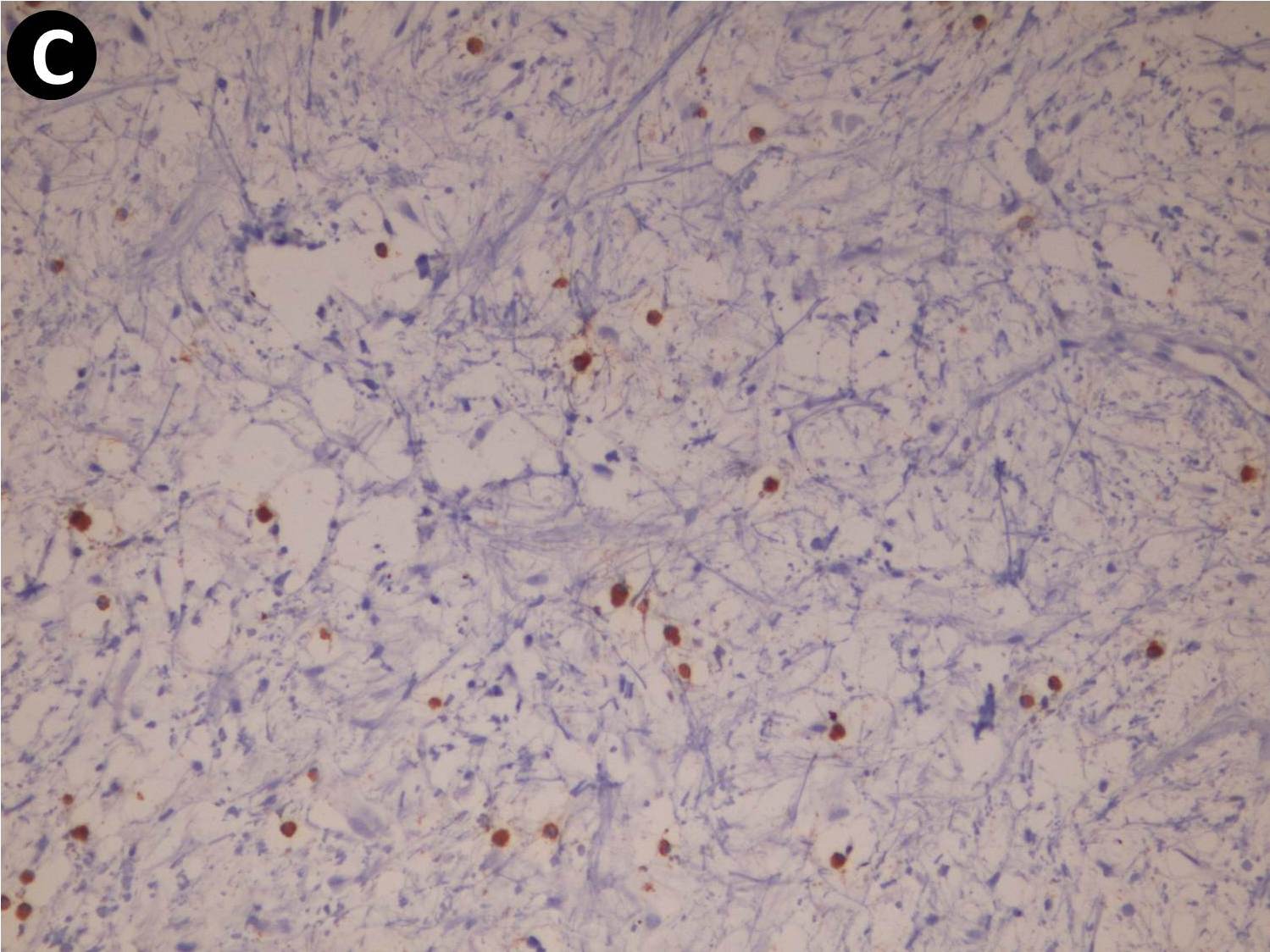
**

**
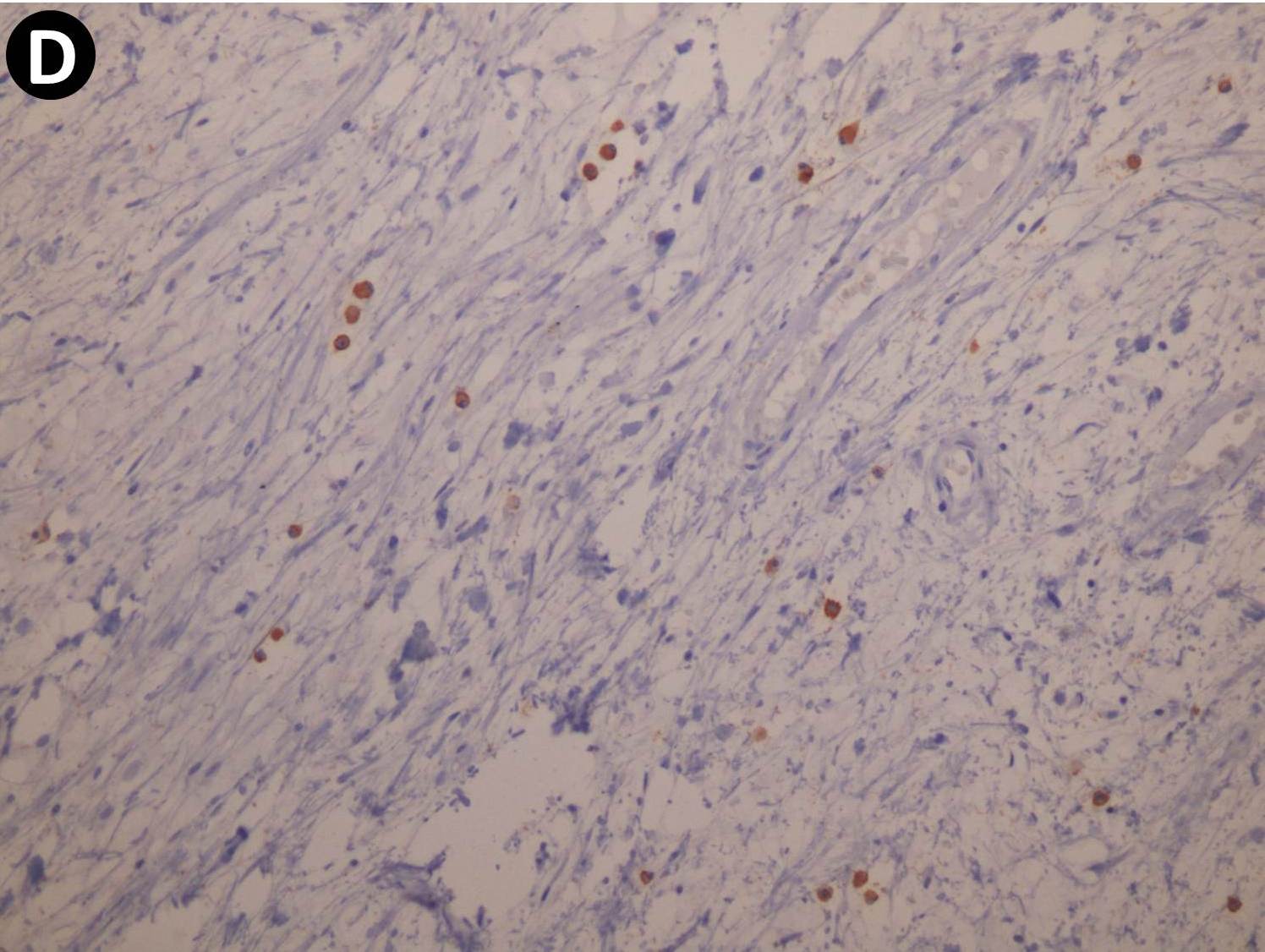
**

**Supplementary Figure 2:** (A) Microscopic section of the tumor showing bland, spindled, and stellate cells with delicate cytoplasmic processes that surrounded blood vessels, on the myxoid stroma (H&E).

(B) Focally positive staining with anti-SMA.

(C) Positive staining with anti-ER.

(D) Positive staining with anti-PR.
