# Supplementary material for: Aggressive angiomayxoma in men: Case report and systematic review
Source: Ann Med Surg (Lond). 2022 Jun 15;79:103880. doi: 10.1016/j.amsu.2022.103880 (PMC9289231; doi:10.1016/j.amsu.2022.103880)
Supplement: Multinedia component 4 [file mmc4.docx]

**Aggressive Angiomayxoma in Men: Case Report and Systematic Review**

| Supplementary Table 1: Summary of Clinical Characteristics of the Patients | | | | | | | | | |
| --- | --- | --- | --- | --- | --- | --- | --- | --- | --- |
| **Characteristics of included studies** | | | | **Patient Characteristics** | | **Clinical Characteristics** | | | |
| Author`s Name | Title | Publishing Year | Patient ID. | Patient No. in the same Article (ex. Case series) | Age (yrs) | Patient Complaints | Physical Examination | Site of the Tumor | Maximal Diameter (cm) |
|  |  |  |  |  |  |  |  |  |  |
| Bégin et al. [4] | Aggressive angiomyxoma of pelvic soft parts: a clinicopathologic study of nine cases | 1985 | 4 | 8 | 20 | Mass |  | Perineum |  |
| Bégin et al.[4] | Aggressive angiomyxoma of pelvic soft parts: a clinicopathologic study of nine cases | 1985 | 5 | 9 | 18 | Polypoid lesion |  | Perineum | 4.5 |
| Tsang et al.[5] | Aggressive angiomyxoma: A report of four cases occurring in men | 1992 | 6 | 1 | 42 | Left scrotal mass over the last 2 months |  | Scrotum | 5 |
| Tsang et al.[5] | Aggressive angiomyxoma: A report of four cases occurring in men | 1992 | 7 | 2 | 68 | Left inguinal mass over the last 3 months | Mass mimicking an inguinal hernia | Groin |  |
| Tsang et al.[5] | Aggressive angiomyxoma: A report of four cases occurring in men | 1992 | 8 | 3 | 70 | Left scrotal swelling over the last 3 months | Mimicking a large epididymal cyst | Scrotum | 14 |
| Tsang et al.[5] | Aggressive angiomyxoma: A report of four cases occurring in men | 1992 | 9 | 4 | 55 | Discomfort in the left ischiorectal fossa over 2 years |  | pelvis | 17 |
| Iezzoni et al.[26] | Aggressive angiomyxoma in males: A report of four cases | 1995 | 10 | 1 | 58 | Painless mass over the last 12 months |  | Scrotum, penis | 14 |
| Iezzoni et al.[26] | Aggressive angiomyxoma in males: A report of four cases | 1995 | 11 | 2 | 45 | Painless mass |  | Scrotum | 3.4 |
| Iezzoni et al.[26] | Aggressive angiomyxoma in males: A report of four cases | 1995 | 12 | 3 | 62 | Mass |  | Scrotum | 6 |
| Iezzoni et al.[26] | Aggressive angiomyxoma in males: A report of four cases | 1995 | 13 | 4 | 51 | Painless mass over the last month |  | Scrotum | 4.5 |
| Murakami et al.[27] | Aggressive angiomyxoma occurring in the scrotum: Report of a case | 1996 | 14 | 1 | 61 | Left scrotal swelling over the last 3 years | No redness, local heat, or spontaneous pain | Scrotum | 25 |
| Hong et al.[28] | Aggressive angiomyxoma of the perineum in a man | 1997 | 15 | 1 | 66 | Worsening symptom of prostate hyperplasia | PRE: soft rubbery lesion inferior to a mildly enlarged prostate | pelves | 6 |
| Layfield et al.[29] | Fine-needle aspiration cytology findings in a case of aggressive angiomyxoma: A case report and review of the literature | 1997 | 16 | 1 | 40 | Large left inguinal palpable nodule |  | Groin | 2.5 |
| Sakata et al.[30] | Aggressive angiomyxoma of the scrotum | 1997 | 17 | 1 | 54 | Right scrotal mass over the last 4 months | Elastic, non-tender and mildly transilluminated mass | Scrotum | 5 |
| Silverman et al.[31] | Comparison of angiomyofibroblastoma and aggressive angiomyxoma in both sexes: Four cases composed of bimodal CD34 and factor XIIIa positive dendritic cell subsets | 1997 | 18 | 1 | 77 | Left spermatic cord mass |  | Spermatic cord | 6 |
| Durdov et al.[32] | Aggressive angiomyxoma of scrotum | 1998 | 19 | 1 | 37 | Left scrotal mass |  | Scrotum | 7 |
| Choong et al.[33] | Aggressive angiomyxoma of the abdominal wall: Previously unrecognised extrapelvic site - Case report and literature review | 1999 | 20 | 1 | 76 | Enlarged right soft loin mass over the last 4 years | Mass in the subcutaneous compartment in the abdominal wall | Abdominal wall | 13 |
| Rhomberg et al.[34] | Aggressive angiomyxoma: Irradiation for recurrent disease | 2000 | 21 | 1 | 27 | Firm subcutaneous left leg mass |  | lower extremity |  |
| Carlinfante et al.[14] | Aggressive angiomyxoma of the spermatic cord. Two unusual cases occurring in childhood | 2001 | 22 | 1 | 13 | Painless cyst |  | Spermatic cord | 9.5 |
| Carlinfante et al.[14] | Aggressive angiomyxoma of the spermatic cord. Two unusual cases occurring in childhood | 2001 | 23 | 2 | 13 | Right scrotal swelling over the last 2 months | Right scrotal swelling with mild bilateral hydrocele | Spermatic cord | 2 |
| De La Ossa et al.[35] | Sonographic appearance of aggressive angiomyxoma of the scrotum | 2001 | 24 | 1 | 26 | Left scrotal mass over the last 2.5 years | Scrotal mass inferior to left testicle | Scrotum | 7.5 |
| Rao et al.[36] | Aggressive angiomyxoma of the epididymis-a case report | 2001 | 25 | 1 | 56 | Left inguinoscrotal swelling over the last year | Non-tender, soft, and transilluminated swelling | Scrotum |  |
| Chuang et al.[37] | Aggressive angiomyxoma of the scrotum | 2002 | 26 | 1 | 24 | Right scrotal mass | Elastic and non-tender mass | Scrotum | 7 |
| Chihara et al.[1] | Aggressive angiomyxoma in the scrotum expressing androgen and progesterone receptors | 2003 | 27 | 1 | 47 | Right soft scrotal mass over the last 2 years | Soft non-transilluminated mass | Scrotum | 17 |
| Kim et al.[38] | Aggressive angiomyxoma of childhood: Two unusual cases developed in the scrotum | 2003 | 28 | 1 | 8 | Left scrotal mass |  | Scrotum | 1.7 |
| Kim et al.[38] | Aggressive angiomyxoma of childhood: Two unusual cases developed in the scrotum | 2003 | 29 | 2 | 1 | Scrotal mass on the 100th day after birth |  | Scrotum | 17 |
| Mermershtain et al.[39] | Aggressive paratesticular angiomyxoma: An unusual locally aggressive benign intrascrotal neoplasm | 2004 | 30 | 1 | 35 | Left painless scrotal mass over the last 7 months | Enlarged left testis without scrotal involvement | Scrotum | 5 |
| Dursun et al.[40] | Aggressive angiomyxoma in a child with chronic renal failure | 2005 | 31 | 1 | 15 | Slow enlargement in the right testicle over the last year | Homogeneous testis with right-sided mass | Scrotum | 4.5 |
| van Roggen et al. [41] | Aggressive angiomyxoma: a clinicopathological and immunohistochemical study of 11 cases with long-term follow-up | 2005 | 32 | 1 | 57 |  |  | Scrotum | 5 |
| van Roggen et al. [41] | Aggressive angiomyxoma: a clinicopathological and immunohistochemical study of 11 cases with long-term follow-up | 2005 | 33 | 2 | 36 |  |  | Groin | 9.5 |
| van Roggen et al. [41] | Aggressive angiomyxoma: a clinicopathological and immunohistochemical study of 11 cases with long-term follow-up | 2005 | 34 | 3 | 44 |  |  | Spermatic cord | 8 |
| van Roggen et al. [41] | Aggressive angiomyxoma: a clinicopathological and immunohistochemical study of 11 cases with long-term follow-up | 2005 | 35 | 7 | 38 |  |  | Perineum | 5 |
| van Roggen et al. [41] | Aggressive angiomyxoma: a clinicopathological and immunohistochemical study of 11 cases with long-term follow-up | 2005 | 36 | 10 | 69 |  |  | Scrotum | 6 |
| van Roggen et al. [41] | Aggressive angiomyxoma: a clinicopathological and immunohistochemical study of 11 cases with long-term follow-up | 2005 | 37 | 11 | 45 |  |  | Pelves | 15 |
| Hidayat et al.[9] | Myxomas and angiomyxomas of the orbit: a clinicopathologic study of 6 cases | 2006 | 2 | 2 | 7 | Proptosis, loss of inferior visual field, decreased visual acuity over the last 9 months |  | Orbit | 2.5 |
| Hidayat et al.[9] | Myxomas and angiomyxomas of the orbit: a clinicopathologic study of 6 cases | 2006 | 3 | 5 | 48 | Palpable mass |  | Orbit | 1.5 |
| Bastian et al.[42] | Aggressive angiomyxoma of the prostate mimicking benign prostatic hyperplasia | 2006 | 38 | 1 | 64 | Symptoms of prostatic enlargement | PRE: unremarkable | Prostate |  |
| Hatano et al.[43] | Rare case of aggressive angiomyxoma presenting as a retrovesical tumor | 2006 | 39 | 1 | 59 | Retrovesical mass discovered accidently through abdominal US | PRE: Soft and smooth mass located on the retrovesical space and isolated from the prostate | Pelvis | 7 |
| Idrees et al.[44] | Aggressive angiomyxoma of male genital region. Report of 4 cases with immunohistochemical evaluation including hormone receptor status | 2006 | 40 | 1 | 43 | Left painless scrotal mass |  | Scrotum | 4.5 |
| Idrees et al.[44] | Aggressive angiomyxoma of male genital region. Report of 4 cases with immunohistochemical evaluation including hormone receptor status | 2006 | 41 | 2 | 68 | Asymptomatic testicular mass over 1 month | Mimicking a left testicular mass | Scrotum | 5.5 |
| Idrees et al.[44] | Aggressive angiomyxoma of male genital region. Report of 4 cases with immunohistochemical evaluation including hormone receptor status | 2006 | 42 | 3 | 81 | Ill-defined mass accidentally discovered during hernia repair surgery |  | Groin | 2 |
| Idrees et al.[44] | Aggressive angiomyxoma of male genital region. Report of 4 cases with immunohistochemical evaluation including hormone receptor status | 2006 | 43 | 4 | 47 | Left scrotal mass |  | Spermatic cord | 6 |
| Andres et al.[45] | Angiomyxoma diagnosed in a man presenting for abdominal lipectomy | 2007 | 44 | 1 | 47 | Accidentally during presenting to abdominal lipectomy |  | Scrotum |  |
| Heffernan et al.[46] | Unusual location of aggressive angiomyxoma in a male | 2007 | 45 | 1 | 76 | Left inguinal painless mass | Large palpable mass over the left proximal thigh anteriorly, extending above inguinal ligament and causing fullness in the left iliac fossa and hip flexion restricted | Groin | 22 |
| Tiwari et al.[47] | Aggressive angiomyxoma of the jejunum: a rare cause of obscure gastrointestinal bleeding | 2007 | 46 | 1 | 35 | Syncope following an episode of melaena | Shock symptoms | Jejunum | 0.5 |
| Wu et al.[48] | Scrotal aggressive angiomyxoma mimicking inguinal hernia | 2007 | 47 | 1 | 40 | Right scrotal painless mass mimicking inguinal hernia | Elastic, non-tender, non-transilluminated, and not reducible scrotal mass | Scrotum |  |
| Hastak et al.[2] | Aggressive angiomyxoma in Men | 2008 | 48 | 1 | 65 | Left perineal swelling over the last 2 years | Freely moveable skin over the swelling without any symptoms | Perineum | 13 |
| Heffernan et al.[49] | Aggressive angiomyxoma of the thigh | 2008 | 49 | 1 | 54 | Asymptomatic femoral mass | Firm, fixed, and non-tender mass | lower extremity |  |
| Pai et al.[50] | Aggressive angiomyxoma of supraclavicular fossa: A case report | 2008 | 50 | 1 | 48 | Right supraclavicular mass over the last year | Firm, non-tender, and fixed nodule | Supraclavicular Fossa | 16 |
| Malik et al. [51] | Aggressive angiomyxoma of the spermatic cord: A rare entity | 2009 | 51 | 1 | 18 | Left hemiscrotal swelling over the last 6 months | Fluctuant, transilluminated, and non-tender, irreducible swelling | Scrotum | 6 |
| Minagawa et al.[52] | Aggressive angiomyxoma mimicking inguinal hernia in a man | 2009 | 52 | 1 | 37 | Left lower abdominal swelling over the last 6 months | Non-tender swelling without redness or local inflammation | Groin | 10 |
| Morag et al.[53] | Aggressive angiomyxoma of the scrotum mimicking huge hydrocele: case report and literature review | 2009 | 53 | 1 | 64 | Scrotal swelling over the last 2 years | Non-tender scrotal swelling with normal testicles | Scrotum | 19 |
| Kondo et al.[24] | Aggressive angiomyxoma in the inguinal region: A case report | 2010 | 54 | 1 | 68 | Inguinal soft swelling over the last 5 years |  | Groin | 7.5 |
| Rehman et al.[54] | Aggressive angiomyxoma of scrotum presenting as an inguinal hernia | 2010 | 55 | 1 | 45 | A large painful right-sided scrotal swelling over the last 4 years | Elastic, tender, irreducible, and non-transilluminated swelling | Scrotum | 13 |
| Sawada et al.[55] | A rare benign genitourinary tumor in a Japanese male: Urinary retention owing to aggressive angiomyxoma of the prostate | 2010 | 56 | 1 | 67 | An intractable nocturia over the last 6 months |  | Prostate |  |
| Sylvester et al.[56] | Aggressive angiomyxoma of larynx: Case report and literature review | 2010 | 57 | 1 | 47 | Dysphonia over the last month | OL (On laryngoscopy): left-sided, mucosa-covered, smooth, supraglottic mass | Larynx | 4 |
| Medina et al.[17] | Aggressive angiomyxoma in men: Clinical case report and literature review | 2011 | 58 | 1 | 58 | Episode of rectal bleeding followed 2 years of growth of right gluteal mass | Soft, ill-defined edges gluteal mass. PRE: firm mass attached to the right side of the rectum without infiltration | Pelvis | 11.5 |
| Rocco et al.[19] | Massive recurring angiomyxoma of the scrotum in an obese man | 2011 | 59 | 1 | 46 | Recurrence of scrotal angiomyxoma | Massive enlargement of the scrotum, the skin was dyschromic, with some decubitus ulcers | Scrotum | 34 |
| Xiao et al.[15] | Aggressive angiomyxoma of the sphenoidal sinus | 2011 | 60 | 1 | 11 | Progressive headache and occasional nasal bleeding over the last 3 years | Purulent secretion in the right middle nasal meatus | Sphenoidal Sinus | 2 |
| Mishulin et al.[57] | Aggressive glabellar angiomyxoma with orbital extension | 2012 | 61 | 1 | 62 | Asymptomatic mass in the right glabella and infra-medial brow over the last 6 months | The nasolacrimal duct was patent | Glabella | 2.5 |
| Gaunay et al.[12] | Aggressive angiomyxoma of the scrotum | 2013 | 62 | 1 | 40 | Scrotal Mass | Ovoid, non-tender, solid, and non-transilluminated mass | Scrotum | 4.2 |
| Marek Karwack et al.[20] | Radiographic diagnosis and differentiation of an aggressive angiomyxoma in a male patient | 2013 | 63 | 1 | 81 | Right perineal swelling for 1 year, defecation problems, feeling of discomfort during prolonged seating | Indolent, soft, and ill-defined perineal lump | Perineum | 15 |
| Saha et al.[58] | Aggressive angiomyxoma of greater omentum with pleural effusion in a young male | 2014 | 64 | 1 | 17 | Abdominal swelling over the last 3 months, dry cough over the last month | Non-tender, firm, diffuse swelling felt all over the abdomen | Greater Omentum | 25 |
| Tyagi et al.[59] | Robotic assisted excision of retrovesical angiomyxoma in a male patient | 2014 | 65 | 1 | 62 | Mild obstructive lower urinary tract symptoms over the last 1 year, with a history of acute retention of urine 3 months back | PRE: cystic mass simulating benign prostatic hyperplasia | Prostate | 6 |
| ALHumoud et al.[60] | Perianal aggressive angiomyxoma in a male patient | 2015 | 66 | 1 | 45 | Painful perianal swelling over the last months, defecation problems | Soft, ill-defined, perineal lump | Perineum | 29 |
| Yaman et al.[61] | Life-threatening angiomyxoma of the larynx | 2015 | 67 | 1 | 52 | Respiratory distress in the outpatient clinic, dysphagia, dysphonia, cough, and obstructive sleep apnea in the supine position over the last 6 months | OL: large smooth surface polypoid, well-circumscribed mass based in the right supraglottic area of the larynx that obstructed most of the view of the rima glottidis | Larynx | 4.5 |
| Caruso et al.[62] | Lessons from an aggressive angiomyxoma unrecognized and treated as rectal prolapse | 2015 | 68 | 1 | 72 | Abdominal pain, Pollakuria | . | Pelvis | 23 |
| Gonzalez et al.[63] | Aggressive angiomyxoma: Imaging findings in 3 cases with clinicopathological correlation and review of the literature | 2015 | 69 | 3 | 75 | . | . | Pelvis |  |
| Mathur et al.[21] | Aggressive angiomyxoma perineum: A rare soft tissue neoplasm in males | 2015 | 70 | 1 | 30 | Perineal swelling for the last 1-2 year | Perineal mass | Perineum | 22.4 |
| Rao et al.[3] | Aggressive angiomyxoma of scrotum presenting as scrotal lymphedema in a case of postoperative carcinoma penis | 2015 | 71 | 1 | 62 | Painless scrotal swelling over the last 6 months | Soft, non-fluctuant, non-transilluminated, and non-tender, irreducible swelling | Scrotum |  |
| Artigas Raventós et al.[64] | Aggressive angiomyxoma. A rare mesenchymal pelvic tumor | 2016 | 1 | 1 | 65 | Rectal tenesmus, dysuria, sensation of perineal weight | Right perineal mass, PRE: protruded laterally to the lumen of the rectum | Pelvis | 26 |
| Draeger et al.[65] | Aggressive Angiomyxoma as a Rare Differential Diagnosis of Enlargement of the Scrotum | 2016 | 72 | 1 | 73 | Post gluteal herpes zoster scrotal lymphedema with consecutive lymphangitis, feeling of heaviness, and a hindrance to mobility | Massive scrotal enlargement. An indolent, indurated, and coarsened scrotal skin | Scrotum |  |
| Haq et al.[66] | Aggressive Angiomyxoma of the Scrotum: A Case Report and Literature Review | 2016 | 73 | 1 | 41 | Painless scrotal swelling over the last 2 years | Thickened, non-fluctuant, non-transilluminated, and non-tender scrotal swelling | Scrotum | . |
| Ismail et al.[67] | Paratesticular aggressive angiomyxoma-a rare case report | 2016 | 74 | 1 | 65 | Right scrotal swelling over the last 6 months | Soft, transilluminated, and nontender scrotal swelling | Scrotum | 11 |
| Ahmed et al.[68] | Aggressive Angiomyxoma of the Penis: The First Case Report in a 9-Month-Old Infant | 2017 | 75 | 1 | 0.75 | Large penis for age | Nodular, firm in consistency, non-tender, and very large penis | Penis | 12 |
| Aydin et al.[69] | Long-standing aggressive angiomyxoma as a paratesticular mass: A case report and review of literature | 2017 | 76 | 1 | 66 | Huge right scrotal mass with mild heaviness | Mobile, non-transilluminated, painless, and soft scrotal mass | Scrotum | 11.5 |
| Damodaran et al.[22] | Aggressive Angiomyxoma Involving Penis and Urethra – A Case Report | 2017 | 77 | 1 | 62 | Enlarging penile and scrotal mass and severe obstructive lower urinary tract symptoms | Enlarged, deformed penis with enlarged scrotum | Scrotum, penis |  |
| Dehuri et al.[70] | Aggressive angiomyxoma in males | 2017 | 78 | 1 | 39 | Left inguinal mass over the last 6 months, initial was painless then became painful |  | Groin | 5 |
| Dehuri et al.[70] | Aggressive angiomyxoma in males | 2017 | 79 | 2 | 50 | Right inguinal swelling over the last 4 months |  | Groin | 7 |
| Gorsi et al.[71] | Aggressive angiomyxoma of transplanted kidney mimicking posttransplant lymphoproliferative disorder | 2017 | 80 | 1 | 44 | Right painful lower quadrant abdominal swelling, low-grade intermittent fever and dry cough over the last 2 weeks, loss of appetite |  | Renal allograft | 10.5 |
| Sharma et al.[72] | Aggressive Angiomyxoma of Inguinoscrotal Region Mimicking Inguinal Hernia: a Case Report | 2017 | 81 | 1 | 53 | Painless inguinal swelling over the last 8 years with rapid progression for 2 months | Non-tender, irreducible, firm, scrotal swelling | Scrotum | 4 |
| Tadepalli et al.[73] | Aggressive angiomyxoma of scrotum in a young male: A rare entity | 2017 | 82 | 1 | 15 | Scrotal swelling over the last 6 months | Diffuse scrotal swelling | Scrotum | 6 |
| Umranikar et al.[74] | Aggressive angiomyxoma of the perineum: a rare presentation in a male with 4 years follow up | 2017 | 83 | 1 | 79 | Painless scrotal mass | Non-tender, rubbery scrotal mass extending into the perineum | Perineum |  |
| Celik et al.[75] | Aggressive angiomyxoma: A rare tumor of male pelvic cavity | 2018 | 84 | 1 | 55 | Constipation, abdominal swelling, lower urinary tract symptoms (LUTS) over the last year | Palpable suprapubic mass, PRE: immobile, rigid mass in the anterior part of the rectum with no relation to the rectum | Pelvis | 12.5 |
| Hsieh et al.[76] | Aggressive Angiomyxoma—Report of a Rare Male Buttock Lesion | 2018 | 85 | 1 | 46 | Asymptomatic left gluteal mass over the last 2 years with discomfort during sitting | Large, soft, compressible, and non-tender left gluteal mass | Perineum | 23 |
| Law et al.[77] | Aggressive angiomyxoma of scrotum in a man: A case report and literature review of 73 cases of aggressive angiomyxoma in men | 2018 | 86 | 1 | 38 | Right scrotal mass over the last year | Firm and enlarged right testis | Scrotum | 10.5 |
| Neyaz et al.[78] | Rare paratesticular aggressive angiomyxoma with negative oestrogen and progesterone receptors in a male patient | 2018 | 87 | 1 | 53 | Left large scrotal swelling over the last 3 years | Firm to soft, non-transilluminated, non-tender, and mobile swelling | Scrotum | 16.5 |
| Shah et al.[79] | Aggressive angiomyxoma of maxilla: A confounding clinical condition with rare occurrence! | 2018 | 88 | 1 | 47 | Slowly growing swelling in the maxillary anterior alveolar and lip region for 1 year | Single, sessile, well-defined, reddish, ulcerated lesion and firm in consistency, mildly tender, slightly bleeder on palpation | maxilla | 4 |
| Zhao et al.[80] | Application of ultrasound in aggressive angiomyxoma: Eight case reports and review of literature | 2018 | 89 | 5 | 64 | Right scrotal swelling over the last 7 months | Large and painless scrotal mass | Scrotum | 10 |
| Zhu et al.[81] | Aggressive angiomyxoma of the prostate: A case report | 2018 | 90 | 1 | 55 | Prostatic enlargement symptoms |  | Prostate |  |
| Kafka et al.[82] | Incidental resection of a scrotal aggressive angiomyxoma mimicking a spermatocele: a case report | 2019 | 91 | 1 | 62 | Left scrotal mass | Painless, mobile, and soft scrotal mass | Scrotum | 7 |
| Serao et al.[83] | Incidental finding of paratesticular aggressive angiomyxoma in a 72-year-old monorchid male | 2019 | 92 | 1 | 72 | Incidentally discovered during regular follow-up of superficial urothelial carcinoma | Abdominal and scrotal examination was equivocal | Pelvis | 6 |
| Tyagi et al.[84] | Prominent swelling on erection: Perineal angiomyxoma as a rare entity | 2019 | 93 | 1 | 44 | Perineal painless swelling | Firm, non-tender, non-pulsatile, irreducible, non-compressible perineal mass | Perineum | 4.6 |
| Addesso et al.[85] | A Large Paraprostatic Mass in A Man: Rare Presentation of Aggressive Angiomyxoma | 2020 | 94 | 1 | 56 | Lower urinary tract syndromes LUTS |  | Pelvis | 7 |
| Kirkilessis et al.[86] | Aggressive angiomyxoma to 57-year old man | 2020 | 95 | 1 | 57 | Scrotal mass | Mobile and non-transilluminated scrotal mass | Scrotum | 11 |
| Korecka et al.[87] | Aggressive Angiomyxoma in an 11-Year-Old Boy - Diagnostic and Therapeutic Dilemmas: An Unusual Case Report and Review of the Literature | 2020 | 96 | 1 | 11 | Right painless scrotal mass | Irreducible, palpated scrotal mass | Scrotum |  |
| Presented Case | | 2021 | 97 | 1 | 72 | Asymptomatic swelling in the right testicle | Hard, moving, painless mass above the right testicle | Scrotum | 6.5 |
| PRE: on rectal examination, OL: on laryngoscopy, yrs: years | | | | | | | | | |
